# Serbian Experience with National Health Accounts

**DOI:** 10.3389/fpubh.2015.00034

**Published:** 2015-02-25

**Authors:** Milena Gajić-Stevanović

**Affiliations:** ^1^Public Health Institute of Serbia “Milan Jovanović Batut”, Belgrade, Serbia

**Keywords:** national health accounts, implementation, health financing, health care reform, health care costs

Serbia, as many countries in Eastern Europe, is in the process of reforming its health system in an effort to improve the efficiency and efficacy of health services and to achieve equal distribution of health resources.

Representatives of Ministry of Health (MOH), Health Insurance Fund (HIF), and Institute of Public Health (IPH) articulated an overall health vision for the health sector in Serbia in August 2002.

In order to get reliable information on financial resources used for health, their sources, and the way they were used, policy-makers realized that they needed a tool that clearly illustrates the flow of funds through the health system. National health accounts (NHA) (1–4), as such a tool, provides a basis for monitoring different health care activities and trends in health spending for all sectors – public and private, providers, diseases, population groups, and regions in country, so Serbian Government have decided to implement the NHA.

Work on development, implementation, and institutionalization of NHA started at the end of 2004, under “Serbia health project,” financed by the World Bank (WB).

The conception of the NHA project was divided in two phases, with following achievements:

In phase I: creation and training of a country team who led the work on the NHA and development of a work plan based on detailed analysis of the situation and assessment of existing data. First NHA have been produced at the beginning of 2006.
In phase II: development of the second round of NHA at the end of 2007 and increased awareness on NHA with eight workshops for different interested structures: policy and decision makers and for institutional experts.

The formation of a new department for NHA production in the Republican Institute of Public Health represents a major accomplishment for the year 2008, after WB project was finished.

National health accounts became an assigned programmatic job of MOH, with the new established financial line for NHA production. Country office of the World Health Organization (WHO) with Dorit Nitzan helped with the implementation process significantly, together with Pia Schneider from WB.

Presenting capability to work on NHA and keep the step with more experienced countries, Serbia has been included in the joint WHO, European Statistics (EUROSTAT), and Organization for Economic Country Development (OECD) work on “A System of Health Accounts (SHA)” version 1.0 into version 11.0 methodology changes. Serbia became participant at OECD, EUROSTAT, and WHO teleconferences, workshops, and OECD internet forum.

From 2008, third and fourth NHA exercises have been accomplished, along with a revision of all previous years’ results, as MOH survey identified discrepancies with Republican Statistical office data on out of pocket payment for health.

Once finished, NHA have produced evidence to help policy makers and health managers to understand health system in Serbia, improve its performance, and better manage health resources (5–13).

Since the first time NHA have been produced, information received was frequently used by Ministry of Health Sector for EU integrations and international Co-operation as a basis for project planning and grants proposals, applications for some study research, presentation of Serbian health system, in country and abroad, like on *Health Policy* Forum *meeting* in Brussels, 2009 along with other types of communication with European commission.

Also, information has been used for:

- Health reform planning, as NHA indicators provide MOH with information needed to review overall health expenditures patterns. In Serbia, NHA revealed that more than 35% of health spending actually came from households, what significantly differed from government official’s perception;
- Assessment of reform achievements, the positive changes are observed like more finances allocation to providers of ambulatory health care, as it was strategically planned;
- Institute of Public Health research purposes, such as analysis of primary health care, cost of illness analysis, assessment of private health sector, Assessment of financial flow in the health system of Serbia in a period 2003–2006, NHA policy impact from 2004 to 2008, etc;
- For development of national strategies, called “Strategy to fight HIV-AIDS from 2011 to 2015”;
- Helping HIV office with UNGASS report;
- Comparison with other countries.

but still poorly used for Ministry of Finance financial projections of a country’s health system requirements.

In 2014, new Health evidence low with specific NHA policy (14) was adopted, as well as Memorandum of understanding between IPH of Serbia, MOH, Ministry of Finance, Republican Statistical Office, and Republican Health Insurance Fund.

National health accounts results are still used mostly for MOH purposes, without broad awareness of NHA existence among Government, civil society, and private sector, presumably as a consequence of lack of financial sources for adequate results dissemination.

## Conclusion and Recommendation

Conclusion and recommendation would be that respective national or international donors should continue to support further work on NHA results dissemination and strategic implementation in some legal document in Serbia at one side, and work with policy makers and managers on use of NHA data on the other side.

Also, the country should work on connection between newly established Central Health Information System and health accounts information.

According to Serbian experience, the biggest reasons for successful NHA implementation and later use of data in the country are the presence of political demand, aid of some donors, and determination of the team who is responsible for NHA institutionalization.

Regional communication and cooperation through workshops and other forms of regional gathering could be the most effective actions in supporting some country in the effort to institutionalize NHA and then communicate NHA results with other countries in region.

National health accounts is expected to be a useful tool for tracing the funds’ flow, for estimating the “health outcome” produced by these funds, and thus for providing detailed input for political decision-taking not only in some region but also all over the world.

## Conflict of Interest Statement

The author declares that the research was conducted in the absence of any commercial or financial relationships that could be construed as a potential conflict of interest.
